# Epidemiological profile of hemophilia A in Karbala-Iraq

**DOI:** 10.25122/jml-2023-0218

**Published:** 2023-11

**Authors:** Inas Muayad Mohammed Ali, Ashwaq Ali Hussein, Israa Mustafa Salih Al-Musawi, Sabeeha Sahib Hadi Hillawi, Naus Salih Kadhim, Abdulkareem Alaiwi Jasim

**Affiliations:** 1 Department of Pediatrics, College of Medicine, University of Kerbala, Karbala, Iraq; 2 Karbala Teaching Hospital For Children, Karbala, Iraq

**Keywords:** hemophilia A, factor VIII, Iraq, Karbala

## Abstract

Hemophilia is an X-linked congenital bleeding disorder caused by a deficiency of coagulation factor VIII (FVIII) (in hemophilia A) or factor IX (FIX) (in hemophilia B) and is one of the most important hereditary conditions in Iraq. The current study tried to provide a glimpse into the epidemiological and clinical status, as well as complications and treatment used for patients with hemophilia A in Karbala, Iraq. This retrospective research was carried out by reviewing the medical records of 90 male patients diagnosed with hemophilia A registered at the Hereditary Blood Disease Center in Karbala Teaching Hospital for children in Karbala, Iraq. The data was collected from May 10, 2023, until June 15, 2023, and included age, severity, type of bleeding, therapeutic approach, chronic viral infections including hepatitis B, hepatitis C, and human immunodeficiency virus (HIV). The median age of the patients was 15 years (18.86±14.42). In this study, one-third of patients' presentations started in the first year of life. The most common type was severe hemophilia A (factor VIII activity < 1%). Consequently, more than half of the patients were treated with factor VIII concentrate. Four patients had hepatitis C, while HIV was confirmed in one patient. The epidemiological or clinical parameters of hemophilia A patients in Karbala seem similar to those in other cities in Iraq. By considering this preliminary data, further comprehensive studies for patients with hemophilia and associated complications in other provinces or cities in Iraq are highly suggested to provide a clear perspective about the prevalence and epidemiological burdens.

## INTRODUCTION

Hemophilia is a hereditary bleeding disorder linked to the X chromosome. For this reason, its manifestation is mostly seen in males, while females are usually carriers. Hemophilia presents as one of two types: type A and type B, in which the affected coagulation factors are VIII and IX, respectively [1]. Based on factor VIII concentration, hemophilia A could be divided into different levels of severity: less than 1 IU/dL leads to a severe form, while 1–5 IU/dL can cause moderate manifestations. In contrast, patients with factor VIII concentrations in the range of 5–40 present mild hemophilia [1, 2]. Bleeding disorders in patients with hemophilia are mostly characterized by spontaneous bleeding in joints (especially knees and elbows) or skeletal muscles, while cutaneous or subcutaneous bleeding is rare [3]. Joint bleeding of any amount could lead to inflammation and an increase in iron concentration in the synovium, which triggers joint damage, leading to angiogenesis and making the affected area more vulnerable to recurrent bleeding [3, 4].

The primary methods of treatment focus on factor VIII purification from plasma, known as plasma-derived factor VIII (pd-FVIII). Advanced techniques introduce recombinant factor VIII (r-FVIII) [1, 5, 6]. The major problems with both types of factor VIII concentrates are due to immunogenicity and factor VIII inhibitor formation. This limitation was the major motivation for advanced research in hemophilia gene therapy [3].

More than 400,000 patients suffer from hemophilia worldwide. Hemophilia A could be encountered in any race or geographical location, and it is estimated to occur in one in every 10,000 births [7, 8]. There is no clear estimation of hemophilia in all provinces of Iraq, but it seems there has been an increase in patients with hemophilia in Baghdad, Iraq, in recent years [7]. The current study tried to provide a glimpse of the epidemiological and clinical status, complications, and treatment used for the patients with hemophilia A in the Karbala province of Iraq.

## MATERIAL AND METHODS

### Study design and study population

This retrospective research was based on reviewing the medical records of 90 male patients diagnosed with hemophilia A registered at the Hereditary Blood Disease Center in the Karbala Teaching Hospital for Children in Karbala, Iraq. There were no exclusion criteria; all patients registered at the center were included in this study. Hemophilia A was confirmed based on previous studies [7, 9].

### Data Collection

Data was collected between May 10, 2023, and June 15, 2023, and included patient age and age at first diagnosis, severity of disease, factor VIII activity, type of bleeding (target joint or polyarticular), therapeutic approach, and chronic viral infection screening for hepatitis B, hepatitis C, and human immunodeficiency virus (HIV).

### Statistical analysis

All data was analyzed using Statistical Package for Social Sciences (SPSS) software version 24. Categorical data were compared using the chi-square test. A p-value of >0.05 was considered statistically significant.

## RESULTS

### Demographical information

A total of 90 male patients with hemophilia A were enrolled in this study. The median age was 15 years (18.86±14.42), 36 (40%) were less than 10 years old, and 57 (63.3%) had a positive family history of hemophilia. One-third of patient presentations started in the first year of life. Fifteen patients (16.7%) and 12 patients (13.3%) had their first presentation and diagnosis in the first 12 and 6 months of their lives, respectively. More information about patients' demographical and clinical data is presented in Table 1.

**Table 1:** Patients' demographical and clinical data

Variable	Group	No. (%)
**Age (years)**	0_10	36 (40%)
	11_20	16 (17.8%)
	21_30	17 (18.9%)
	31_40	12 (13.3%)
	41_50	6 (6.7%)
	>50	3 (3.3%)
**Family history**	Positive	57 (63.3%)
	Negative	33 (36.7%)
**Severity**	Mild	17 (18.9%)
	Moderate	16 (17.8%)
	Severe	57 (63.3%)
**Regime of treatment**	On demand	53 (58.9%)
	Prophylaxis	37 (41.1%)
**Type of** **treatment**	Factor VIII only	47 (52.2%)
	Factor VII	9 (10%)
	Factor VIII & cryoprecipitate	30 (33.3%)
	Factor VIII & cryoprecipitate & emicizumab	4 (4.4%)
**Inhibitors**	Yes	9 (10%)
	No	81 (90%)
**Target joints**	Yes	46 (51.1%)
	No	44 (48.9%)
**Viral infection**	Yes	5 (5.6%)
	No	85 (94.4%)
**Residency**	Karbala	85 (94.4%)
	Other Governorate	5 (5.6%)
**Total**		90 (100%)

### Clinical characteristics

**Figure 1:**
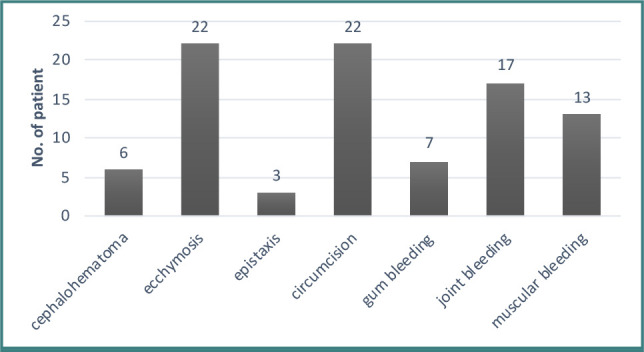
Distribution of patients according to site of bleeding in first-time of presentation

Patients with hemophilia commonly present with bleeding after circumcision (22 patients), ecchymosis (22 patients), and joint bleeding (17 patients) (Figure 1). Seventeen patients (18.9%) showcased mild hemophilia A, 16 patients (17.8%) had moderate hemophilia A, and 57 patients (63.3%) presented with the severe type. Patients with severe hemophilia commonly present primary symptoms in the first year of life.

The number of patients with target joint involvement was 46 (51.1%), while there were 25 (54.3%) patients with multiple joint involvement. Target joint involvement and age at initial presentation were positively correlated (p-value=0.049), as seen in Table 2.

**Table 2:** Distribution of target joint involvement in patients with hemopilia according to the age at the primary presentation

Age at the primary presentation	No.	Target joint involvement only No.	p-value
**0-6 months**	29	19	0.049
**6-12 months**	29	15	
**12-24 months**	12	6	
**2-18 years**	17	6	
**More than 18 years**	3	0	

p-value<0.05 was considered statistically significant

### Therapeutic evaluation

About two-thirds (58.9%) of patients with hemophilia received on-demand therapy, while 41.1% received prophylaxis treatment. Treatment regime and target joint involvement were positively correlated (p-value=0.0001), as seen in Table 3.

**Table 3:** Distribution of target joint involvement in patients with hemopilia according to the regime of treatment

Regime of treatment	No.	Target joints involvements No.		p-value
		Yes	No	
**On demand**	53	37	16	0.0001
**Prophylaxis**	37	9	28	

p-value<0.05 was considered statistically significant

More than half of the patients were treated with factor VIII concentrate (n=47, 52.2%), while others received cryoprecipitate. During the evaluation, nine (10%) patients had inhibitors against the clotting factors.

There was no statistically significant link between inhibitor formation and treatment (p-value=0.421), as seen in Table 4.

**Table 4:** Distribution of inhibitor formation in patients with hemopilia according to the regime of treatment

Regime of treatment	No.	Inhibitors No.		p-value
		Yes	No	
**On demand**	53	4	49	0.421
**Prophylaxis**	37	5	32	

p-value<0.05 was considered statistically significant

### Chronic viral infections

Four patients had hepatitis C infection (4.4%) while only one patient had HIV (1.1%). There were no hepatitis B virus positive patients in this study. There was no statistically significant link between primary presentation age and chronic viral infection (p-value=0.058) (Table 5).

**Table 5:** Distribution of chronic viral infection in patients with hemopilia according to the age at the primary presentation

Age at the primary presentation	No.	Chronic viral infection No.	p-value
**0-6 months**	29	3	0.058
**6-12 months**	29	2	
**12-24 months**	12	0	
**2-18 years**	17	0	
**More than 18 years**	3	0	

p-value<0.05 was considered statistically significant

## DISCUSSION

While there are some reports of hemophilia prevalence and epidemiological burdens in Iraq, there are no unifying country-wide studies focused on this subject [7, 10, 11]. By evaluating 90 male patients with hemophilia A, this research provided insights into the epidemiological, clinical status, complications, and treatment of Iraqi patients in the Karbala province.

In this study, one-third of patient presentations started in the first year of life. The most common type was severe hemophilia A, and more than half of patients were treated with factor VIII concentrate. Four patients had hepatitis C infection, while HIV was confirmed in one patient.

In a study on patients with hemophilia A in Al-Ramadi City, conducted by AL-Zubaidy *et al*. [10], 57.8% of the patients were less than 20 years old, similar to the results of Erbil *et al*. [12]. A study by Lateef *et al*. showcased that 50% of patients get diagnosed in the first year of life, similar to our results [11].

Lateef *et al*. [11], also showcased an 11% prevalence of severe hemophilia A in their sample. Meanwhile, Taresh *et al*. [13] study represents 39.8% of patients with severe hemophilia. This finding is mostly consistent with our current data. The only major difference among these studies is the number of patients with severe hemophilia, possibly due to a limited number of eligible patients.

Lateef *et al*. [11], in a study conducted in Diyala, showcased a 64% prevalence of joint affections. Other research conducted throughout Iraq had reported different figures for target joint involvement [7, 12]. Our current study displays a 54% prevalence of target joint involvement. Despite minor differences, most research highlights a similar prevalence. Our research, as well as similar studies conducted throughout Iraq, provides findings with reasonable divergences about the family history of this affection [11, 14]. The main treatment given to our patients was recombinant factor concentrate, and about two-thirds of them received it on demand. This result is approximately similar to the that of Lateef *et al*. [11], and Kadhim *et al*. [7].

In our current study, 11.6% of patients had inhibitors against clotting factors during treatment with factor VIII. Other studies in Iraq and Jordan present the same prevalence of inhibitors in patients with hemopilia receiving factor VIII (ranging from 11–18%) [13, 15]. In contrast, Dorgalaleh *et al*. report a low prevalence of inhibitors in Iranian patients [16]. This controversy could be associated with sample size or geographical differences. Further studies about the inhibitor prevalence in Iraq are urgently required due to the importance of this matter in the patient’s therapeutic condition.

In a study by Lateef *et al*. [11], hepatitis C virus (HCV) infection was found in 12.5% of patients. Studies about HCV prevalence in patients with hemopilia showcase a 19% HCV prevalence in Baghdad [17].

Also, a higher HCV prevalence in patients with hemopilia in Baghdad was reported by Al-Beldawi *et al*. [18]. In contrast, we found that only 4.4% of patients had HCV infection. This low prevalence of HCV in our current study could be due to the geographical variables. Furthermore, the mean age of the patients with hemopilia included in all three mentioned studies was higher than that of our patients.

The major limitation of our research regards the number of patients included.

## CONCLUSION

Epidemiological or clinical parameters of patients with hemopilia A in Karbala seem similar to those in other cities in Iraq. By considering this preliminary data, further comprehensive studies for patients with hemopilia and associated complications in other provinces or cities in Iraq are highly recommended to provide a clear perspective on hemophilia prevalence and epidemiological burdens. Patients with hemopilia with complications have a heavy impact on the family and society, so the improvement in comprehensive care can ensure longer life expectancy and increased quality of life for patients. Awareness, education, and genetic counseling are needed to prevent the spread of such disorders in the community.
